# Beta-adrenergic antagonist for the healing of chronic diabetic foot ulcers: study protocol for a prospective, randomized, double-blinded, controlled and parallel-group study

**DOI:** 10.1186/s13063-020-04413-z

**Published:** 2020-06-08

**Authors:** Ramanjot Kaur, Catherine Tchanque-Fossuo, Kaitlyn West, Yasmin Hadian, Anthony Gallegos, Daniel Yoon, Ligia Ismailyan, Saul Schaefer, Sara E. Dahle, R. Rivkah Isseroff

**Affiliations:** 1 Dermatology Service, VA Northern California Health Care System, Mather, CA USA; 2 Department of Dermatology, UC Davis Medical Center, Sacramento, CA USA; 3 Podiatry Service, VA Northern California Health Care System, Mather, CA USA; 4 Department of Internal Medicine, UC Davis Medical Center, Sacramento, CA USA

**Keywords:** Diabetic foot ulcer, Chronic wounds, Nonhealing wounds, Timolol, Randomized controlled trial

## Abstract

**Background:**

Diabetic foot ulcers (DFUs) are the most common cause of leg amputations and their management is extremely challenging. Despite many advances and expensive therapies, there has been little success in improving outcomes of DFUs. In prior work our laboratory has examined the effects of beta-adrenergic antagonists (βAAs) on skin and skin-derived cells. We have shown that βAAs enhance the rate of keratinocyte migration, promote angiogenesis, and hasten wound healing in scratch wounds in vitro, in animal wound models, and in anecdotally reported cases of chronic wounds that healed successfully after topical application of the βAA timolol. Thus, we propose to test timolol directly on DFUs to determine if it improves healing above the current standard of care (SOC). This study will examine the efficacy and safety of topically applied beta-antagonist Timoptic-XE® (timolol maleate ophthalmic gel forming solution) in subjects with DFUs.

**Methods/design:**

This is a phase two, randomized, double-blinded, controlled, and parallel-group clinical trial with two treatment arms, SOC plus topical Timoptic-XE® and SOC plus a non-biologically active gel (hydrogel, as placebo drug). Study subjects with a DFU will be selected from the Veterans Affairs Northern California Health Care System (VANCHCS). Study duration is up to 31 weeks, with three phases (screening phase for two weeks, active phase for up to 12 weeks, with an additional second consecutive confirmatory visit after 2 weeks, and follow-up phase comprising monthly visits for 4 months). Subjects will apply daily either the topical study drug or the placebo on the foot ulcer for 12 weeks or until healed, whichever comes first. Measurements of wound size and other data will be collected at baseline, followed by weekly visits for 12 weeks, and then a monthly follow-up period.

**Discussion:**

This is a clinical translation study, moving the investigators’ pre-clinical laboratory research into a translational study in which we will analyze clinical outcomes to assess for safety and estimate the efficacy of a topical beta-antagonist in healing of DFUs. The results from this trial may establish new treatment paradigms and safety profile for DFU treatment.

**Trial registration:**

ClinicalTrials.gov, NCT03282981. Registered on June 14th, 2018.

## Background

Diabetic foot ulcers (DFUs) account for significant morbidity and immense biomedical burden. Aside from the management of multiorgan comorbidities in diabetic patients, DFUs alone substantially impact our health care system with both economic and psychosocial effects. A diabetic patient has a 25% lifetime risk of developing a DFU [1]. As the DFU becomes intractable, the patient’s quality of life and productivity are considerably affected [1–3]. There is a one in six risk that patients with a DFU will have an amputation, with a 66% risk of recurrence and a 47% increase in mortality, as high as colon cancer [3–5]. Notably, according to the US Census Bureau, there are 21.8 million veterans in the US, and nearly 25% have diabetes compared to 10.5% of the general population [6–10]. A study involving veterans with diabetes and a foot ulcer noted that there was a 2.39 increased relative risk of death compared to those without a foot ulcer [11]. Preventative measures are taken to reduce the burden of diabetes and aggressive treatment is used for the ulcers before they advance to amputation.

Health care systems invest enormous sums of money in improving the healing of DFUs, with an estimated annual cost for DFU treatment in the US exceeding $10.9 billion [12]. Several advanced treatments for DFUs exist, but they are expensive, difficult to use in the clinic or at home, and have shown limited success in the healing of DFUs. The cost per episode of DFU treatment can easily exceed $38,000 plus associated expenses related to hospitalization, ulcer recurrences, amputations, home health care, dressings, decreased productivity and premature disability, social isolation, and depression [1–3, 8, 13].

The standard of care (SOC) for a DFU generally consists of the debridement of necrotic tissue, application of a moist dressing, and the use of offloading devices (such as foot orthoses, total contact cast and/or with the use of crutches, wheelchair, scooter, or other assistive ambulatory devices) that protect the wound from pressure or trauma related to ambulation and other acts of daily living, and management for infection if indicated [2, 3, 13–18]. Nevertheless, despite wound specialists adhering to the best standard wound care regimens, only 31% of DFUs heal after 20 weeks of care [18, 19]. Such an unfavorable cure rate has prompted increased research for therapeutic alternatives and novel approaches to optimize wound healing, particularly for the growing Veterans Affairs (VA) diabetic population. Therefore, preventing further complications and the high costs associated with DFU treatment is critical. Thus, we are investigating using a safe, inexpensive, well-characterized, easy to use drug that could be implemented system-wide to enhance DFU repair and may have far-reaching consequences.

In prior work our laboratory has examined the effects of beta-adrenergic antagonists (βAA) on skin and skin-derived cells. Of the several classes of adrenergic receptors, predominant expression of the β2 subtype has been identified on major cell types of the skin, including human keratinocytes, melanocytes, and dermal fibroblasts [20–23]. Keratinocytes can also synthesize catecholamines such as epinephrine and norepinephrine [20–24], in essence creating a self-contained, catecholamine signaling network. The functional role of this network has been elusive. Our work suggests that it contributes to the control of cell migration, and thus to skin wound healing [25–28].

When skin is wounded, repair mechanisms are activated to restore skin integrity. The repair process involves the orchestration of interactions between cellular components, growth factors, chemokines, and extracellular matrix proteins that regulate the migration and proliferation of the keratinocytes into the wound [29–31]. This directional migration of keratinocytes into the wound is critical for repair and reestablishment of epithelial coherence. Our laboratory work has shown that activation of βAR by stress-catecholamine agonists, such as epinephrine and norepinephrine, decreases keratinocyte migration, decreases the ability to heal an in vitro scratch wound, and impairs healing of acute wounds in animal models [23, 27, 28, 31–34]. Importantly, these βAR agonists are found in significant concentrations in human DFU tissues [28].

The natural corollary to the finding of stress catecholamine βAR ligands within the wound environment that can impair pro-reparative functions of skin-derived cells [23–28, 30, 31, 35] was to determine the effects of blocking their action. Importantly, and specifically relevant to our proposed clinical trial, we have shown that blockade of the βAR with antagonists improves healing in vitro and in animal models [24, 26, 28]. Work by other investigators also supports the hypothesis that βAR antagonists can improve DFU healing. Collagen synthesis (in a pulmonary injury model) has been observed to be increased by βAR antagonists [36]. More specifically, Gulcan and colleagues demonstrated that the topical application of βAR antagonists to wounds in diabetic rats improves not only the rate of healing, but wound vascularity [37]. Indeed, a patent application on the use of a βAR antagonist for the healing of DFUs has been filed by other investigators [38], albeit with no human clinical data. Our goal with this clinical trial is to generate these unequivocal data and to demonstrate the safety and efficacy of this approach to improve healing in human chronic DFUs.

Here we propose a clinical translational study, moving our original laboratory research into an early clinical trial to test this hypothesis. Therefore, the main aim of this proposal is to establish timolol ((S)-1-[(1,1-dimethylethyl)amino]-3-[[4-(4-morpholiny)-1,2,5-thiadiazol-3-yl]oxy]-2-propanol (Z)-2-butenedioate(1:1) (salt)), a non-selective beta-adrenergic antagonist, as a novel therapeutic alternative in response to the challenging clinical management of DFUs. We expect this study will demonstrate that timolol, a low-cost therapy, improves the rate of wound healing, which in turn will have tremendous long-term benefits, improving morbidity, quality of life, as well as the negative psychological and social issues associated with DFUs.

## Methods/design

### Design

We propose a phase 2, randomized, double-blinded, controlled, and parallel-group clinical trial to assess the effectiveness of topical timolol (SOC plus topical Timoptic-XE®) compared to standard of care (SOC; plus a non-biologically active gel, hydrogel) on DFUs.

#### Study objectives

Our primary objective is to test the hypothesis that topically applied timolol can significantly increase complete ulcer healing within 12 weeks.

Our secondary objective is to assess the safety profile of topically applied timolol in the treatment of DFUs. Building on the excellent safety record of timolol in various topical applications, we will measure the timolol plasma levels during the treatment phase and the rate of adverse events in the setting of a randomized controlled trial.

Other secondary objectives are to measure the following: comparison of two study arms for percentage difference in change in size from randomization visit to the endpoint visit (post 12 weeks), the time to wound closure between the two groups, and wound healing rates in comparison with wound size between the treatment groups; in addition, quality of life using Veterans Rand (VR-36) Health Survey and the Lower Extremity Functional Scale, and the measurement of all the adverse events associated with the use of timolol.

### Study description

#### Study population

This study will be conducted exclusively on veterans who are 18 years of age or older, with a documented diagnosis of diabetes and foot ulceration that has been present for at least 4 weeks. The study protocol is approved by both the Veterans Affairs’ Research and Development Committee and their Institutional Review Board (IRB). The study site is at the main campus, located at the Sacramento VA Medical center, which houses a comprehensive multi-specialty wound clinic. We will recruit patients from this wound clinic as well as from the other six surrounding satellite clinic sites of the VA Northern California Health Care System (VANCHCS). The multi-specialty wound clinic is staffed by dermatologists, podiatrists, a wound/ostomy nurse, and a vascular interventional radiologist. All recruited patients will be seen at this single, multi-specialty wound clinic which treats a variety of wounds, including diabetic, venous, and pressure ulcers. With approximately 1002 patients treated for DFUs in the past year at VANCHCS alone, we have a strong clinical base from which to recruit for this study. This does not include referrals that we anticipate from the primary care, vascular, podiatry and dermatology clinics from the six other satellite clinics within VANCHCS.

The study will consist of volunteer patients who have diabetes mellitus documented using the criteria of the American Diabetes Association, who have a foot ulcer below the malleolus. The DFU must be at least 4 weeks old with a surface area between 0.5–20 cm^2^ after debridement, with no active infection, including cellulitis or osteomyelitis, as listed in Table 1. Subjects who meet the inclusion and exclusion criteria will be randomized to either group A (βAR antagonist group plus SOC) or group B (non-biologically active gel plus SOC). Patients in either group will receive the once-daily application of the study drug for 12 weeks or until the ulcer heals, whichever comes first.

**Table 1:** Inclusion and exclusion criteria

**Inclusion criteria**	
● Have diagnosis of diabetes mellitus	
● Male or female subject of any race aged 18 years or older	
● Lower extremity ulcer located anywhere on the foot up to the ankle	
- Of more than 30 days duration and less than 2 years duration (medically documented)	
- Surface area between 0.5cm^2^ and 20cm^2^ (as measured with the Silhouette imaging system at randomization). The ulcer with largest surface area meeting inclusion criteria will be selected as the index ulcer	
- If two ulcers are present with the same surface area, the ulcer of the longest duration will be selected as the index ulcer	
● Documented ankle–brachial index (ABI) between 0.8 and 1.2 on the study limb or toe pressure over 65 mmHg within 6 months of screening phase	
● Documented biopsy report to rule out malignancy of ulcer of > 6 months’ duration	
**Exclusion criteria**	
● Ulcer of non-diabetic etiology, such as venous, arterial, and burn wounds	
● Index ulcer is less than 3 cm in distance from any other ulcer on the same extremity	
● There are more than three ulcers on the study foot	
● Index ulcer presents with any of the following: cellulitis, osteomyelitis, exposed bone, tendon or fascia, purulent exudates, or gangrene	
● Index ulcer shows evidence of infection (defined as a moderate or severe rating of all of the following clinical signs/symptoms: 1) increased warmth, 2) increased pain, 3) erythema, and 4) malodorous exudate at screening or at randomization (visit 1), OR total organism count > 1 × 10^5^ colony forming units (CFU) from the screening visit study ulcer culture sample)	
● Index ulcer surface area has decreased or increased > 40% between screening and at randomization (visit 1) as assessed by the Silhouette imaging system	
● Has medically documented history of HIV	
● Has active malignancy on the study limb	
● Has uncontrolled diabetes mellitus as defined by glycosylated hemoglobin A1C > 12% within 3 months of screening	
● Has immunodeficiency as defined by serum IgG, IgA, and IgM less than one-half the lower limit of normal	
● Has severe protein malnutrition as defined by serum albumin < 2.5 g/dL	
● Has chronic renal insufficiency requiring dialysis	
● Has serum aspartate aminotransferase (AST, SGOT, GOT) or serum alanine aminotransferase (ALT, SGPT, GPT) levels greater than twice the upper limit of normal	
● Has fatigue, palpitations, dyspnea, and/or angina at rest	
● Has a medically documented or self-reported history, within the previous 12 months from date of screening visit, of alcohol or drug abuse, particularly methadone or heroin	
● Has received previous treatment with the following during the 60 days prior to screening: immunosuppressive agents, radiation, chemotherapy, growth factors (epidermal growth factor, tumor necrosis factor, transforming growth factor, platelet derived growth factor, etc.) at the site of the study ulcer, split- or full-thickness skin graft at the site of the study ulcer, biologically active (or engineered) cellular or acellular product(s) at the site of the study ulcer, investigational drug or device	
● Has been hospitalized for treatment of a diabetic foot ulcer within the previous 30 days from screening	
● Has history of bradycardia (heart rate less than 60)	
● Has ESR > 70 mm/h and CRP > 100 mg/L at time of screening	
● Has medically documented history of hypotension/orthostatic hypotension and/or symptomatic hypotension (systolic blood pressure below 90 and diastolic blood pressure less than 60). (Note that there is no standard testing regimen protocol for orthostatic hypotension, even for patients starting on oral timolol)	
● Currently taking asthma or COPD medications (as documented in chart)	
● Has a medically documented diagnosis of myasthenia gravis, untreated hyperthyroidism, type 1 and/or type 2 heart block	
● Female who is pregnant or refuses to use adequate contraceptive methods and is of childbearing age during the trial	
● Prisoners, institutionalized individuals, or vulnerable population	

#### Study framework

**Fig. 1:**
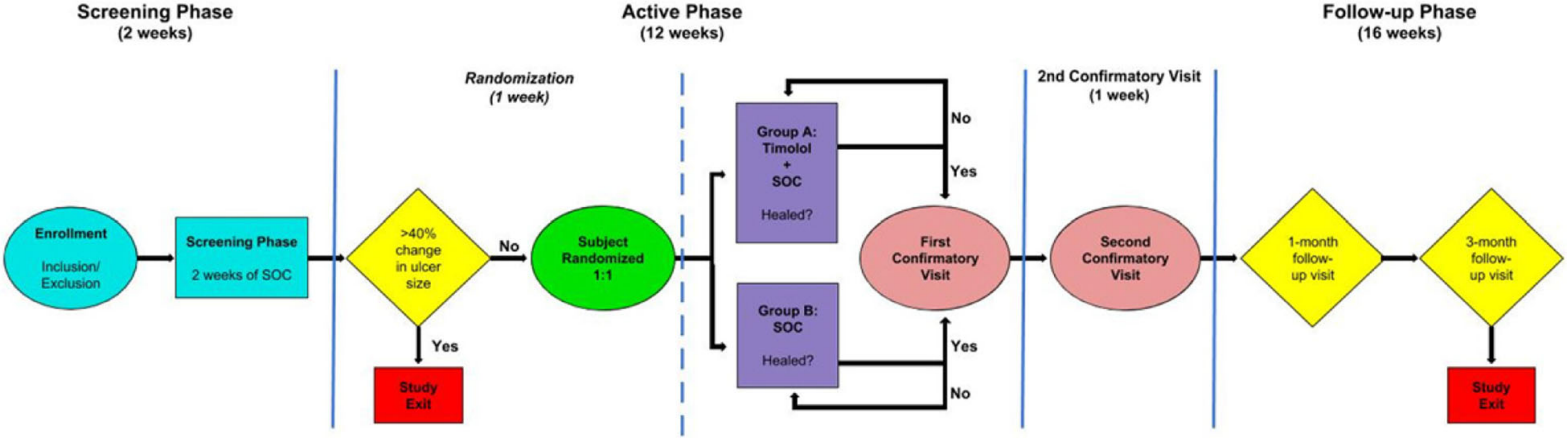
Study timeline

**Fig. 2:**
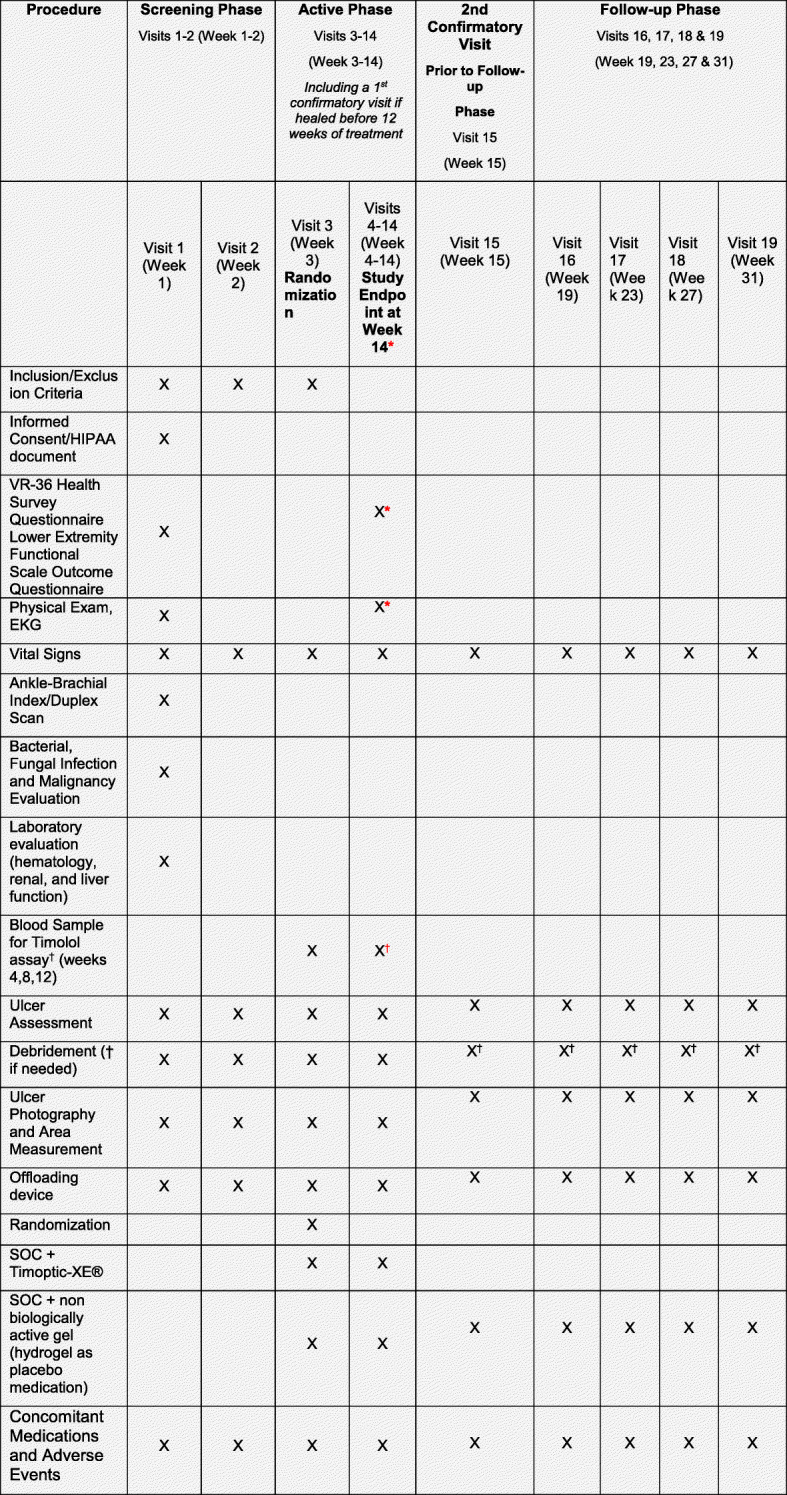
Study diagram

The maximum study duration is 31 weeks, with three phases, described in Fig. 1. The initial two weeks (visits 1–2/weeks 1–2) will consist of the screening phase, followed by 12 weeks (visits 3–15/weeks 3–15) of the active phase. If the DFU wound heals, the active phase also includes two confirmatory visits for 2 weeks. The final phase, follow-up phase, will consist of monthly clinic visits for 4 months (visits 16–19/weeks 19–31; Fig. 2).

**Fig. 3:**
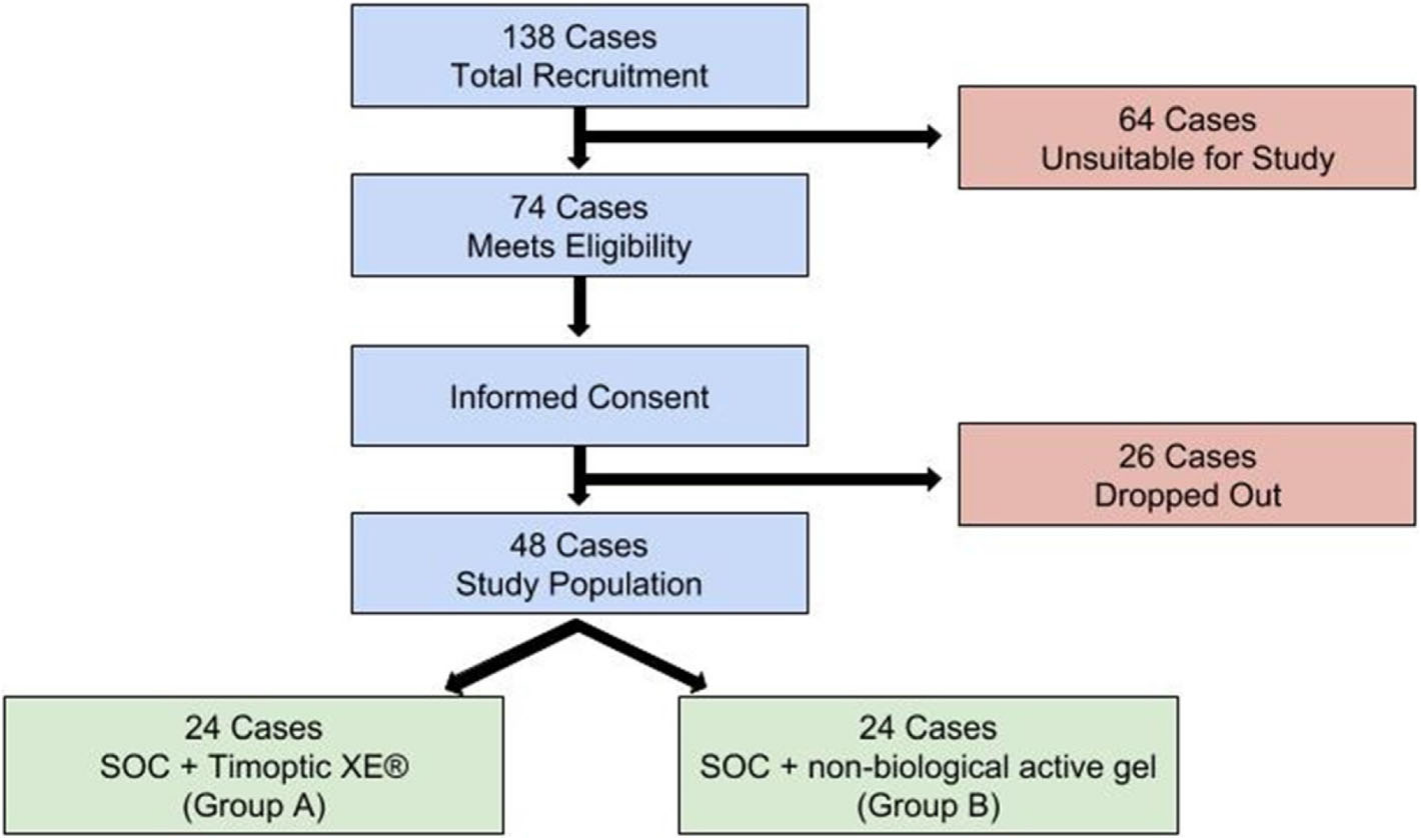
Summary of selection process

The study is powered to determine outcomes with 138 patients to accommodate anticipated withdrawals to achieve enrollment of 48 patients, 24 per arm. Figure 3 illustrates the selection process with the expected number of participants in the study. With a minimum of 60% participation, there should be adequate recruitment for this study. Thus, we will accrue a total target sample of approximately 138 subjects. Of these subjects, we estimate an overall 35% withdrawal/dropout rate for a total of 48 enrolled subjects (24 patients per arm). This includes the presumed 10% of enrolled subjects who will be exited from the study prior to randomization, the 10% who will not meet the primary endpoint analysis inclusion requirement, and the 15% who will not complete the study due to other factors such as treatment failure, loss to follow-up, adverse events, clinical/safety issues, and/or non-compliance.

#### Data collection

The research team, including the principle investigators, will gather the data. Ulcer area measurements will be conducted using the Silhouette® Aranz 3D-digital photography system, pre- and post-debridement of ulcers, along with physical examination and patient documentation of each research visit.

Ulcer measures will be collected at baseline and weekly after that until the completion of the study. The primary endpoint will be obtained at visit 15/week 15, and the final secondary endpoint measurement collected at visit 19/week 31. The area of the target ulcer will be summarized by treatment group and research visits. Both actual value and change of the ulcer area from the previous visit will be calculated, as will changes from baseline, which quantify the weekly changes and the weekly percentage change in the target ulcer area from visit to visit.

Primary analyses will evaluate ulcer healing rates, defined as “skin re-epithelialization without drainage and dressing requirement” by week 12 (end of the active phase of the study). The secondary analyses to evaluate the association of specific wound characteristics (such as change in wound size over time, wound location) and subject characteristics (body mass index, chronicity of the wound, years of diabetes diagnosis, HbA1c, and ABI/toe pressure) will be conducted using logistic regression. Additional logistic regression analysis will be applied to investigate the relationship between the occurrence of each type of adverse event and treatment in order to adjust for each of the potential confounding factors previously listed. A two-way analysis of variance (ANOVA) with a post hoc test will be used to determine the relationship between timolol serum levels and wound healing. For demographic and clinical characteristic data at baseline, continuous variables will be summarized (when appropriate) by using mean, median, standard deviation, co-efficient of variation, minimum value, and maximum value. Categorical variables can be summarized with frequency tables. Baseline comparability between the two treatment groups will be assessed using the independent two-sample *t*-test or Wilcoxon rank-sum test and the Chi-square test or Fisher’s exact test. Finally, we will use ANOVA to analyze quality of life (QOL) using the VR-36 Health Survey, the Lower Extremity Functional Scale outcome [39], and the Charlson Comorbidity Index [40]. For the analyses of the large number of multiple secondary outcomes, *p*-value adjustments will be performed by the procedure of Benjamini and Hochberg [41].

#### Standard of care

The weekly visits for all subjects (treatment and control groups) will consist of ulcer assessment, debridement or removal of necrotic/infected tissues, wound cleansing, dressing to maintain a moist wound environment, and management of wound infection. Ulcer area is to be measured using 3D-digital photography, Silhouette® Aranz camera, before and after sharp debridement of the ulcer. We will use offloading devices (such as orthotics, total contact casts, crutches, wheelchairs) to protect the wound from pressure or trauma relating to ambulation or other daily activities. Treatment for infection will be initiated if it is indicated. The weekly wound assessment is detailed in Table 2. During the initial workup, subjects will be assessed for adequate blood circulation with the ankle–brachial index (ABI), and we will establish and provide nutritional support, which includes blood glucose control that would meet the criteria of the Food and Drug Administration (FDA) Guidance for Industry Chronic Cutaneous Ulcer and Burn Wounds as well as the International Best Practice Guidelines for wound management in DFUs [3].

**Table 2:** Weekly assessment for all study participants

Ulcer assessment and measurement	
- Consistency of wound edges, fibrin, peri-wound erythema, peri-wound edema, peri-wound induration, qualitative quality of foot/leg edema, granulation tissue, necrotic tissue (amount), exudate (type), exudate (amount), wound size (cm^2^), pain and presence of epithelialization	
Lab tests per protocol	
Sharp debridement	
Wound cleansing and moist wound healing dressing	
Digital photograph using the Silhouette Aranz camera	
- Device that provides three-dimensional measurement and documentation of the wound surface area, depth and volume, along with storage and wound informatics management, i.e., graphic depiction of wound progression timeline	
Infection/ osteomyelitis assessment if indicated with confirmatory bacterial culture	
- Deep swab curette, tissue specimen/biopsy), radiographs and blood work (CBC, ESR, CRP, and chemistry). Note: if radiograph suggests but does not confirm osteomyelitis, then follow-up studies of bone scan, bone biopsy, or MRI or CT imaging will be obtained as deemed necessary by investigator clinician	
Use of an offloading device that protects the wound from pressure or trauma related to ambulation and other acts of daily living	
- The total contact cast or instant total contact cast would be ideal offloading devices. However, given the nature and complexities associated with DFU, it is unrealistic to expect that all patients will tolerate such offloading devices. Thus, the offloading device provided will be dependent on the subject’s ability to tolerate the specific offloading device. Adherence to offloading device will be evaluated by the investigator at each visit by observing plantar wear patterns and inquiring from the patient if offloading was used consistently as instructed. Alternative to total contact cast or instant total contact cast (camwalker), offloading devices will be offered including, but not limited to, the use of felt/foam adhesive/post op shoe, custom offloading insole, and customized healing shoe in combination with gait assistive devices such as a roll-a-bout scooter, walker, wheelchair, or motorized scooter.	
Smoking cessation counseling	
Note: Blood glucose monitoring/management will be addressed by the consultant endocrinologist for any patient with HbA1C above 8	

Group A: SOC plus Timoptic-XE®. The subjects will receive the SOC treatment as described above plus Timoptic-XE®

Group B: SOC plus non-biologically active gel (hydrogel, as placebo medication)

All other procedures are as in the SOC group

#### Specifications of study drug: Timoptic-XE®

Timolol has been used since the 1970s as a systemic blood pressure lowering agent and has a strong safety profile. It is a non-selective, reversible, beta-adrenergic receptor blocker [42]. Its uses include systemic treatment of hypertension, angina pectoris, cardiac arrhythmias, migraine, and the reduction of mortality following myocardial infarction [43]. It is also widely used as an ophthalmic solution in the treatment of glaucoma to reduce intraocular pressure [44, 45]. Topically applied, timolol has also shown great success with a good safety profile as adjunct therapy for infantile hemangiomas in several countries around the world [46–54]. In fact, the FDA has now approved the use of a similar beta blocker, propranolol, as an oral pediatric formulation of propranolol hydrochloride (Hemangeol) for proliferating hemangioma [55, 56]; currently it is the most common form of therapy for children with ulcerated hemagiomas [46–54, 56]. The drug to be used in this study is the commercially available ocular formulation of timolol, timolol maleate ophthalmic gel forming solution, which has been developed as an extended release preparation (Timoptic-XE®, Merck & Co, Inc.). In addition, the FDA has provided our investigative team with Investigational New Drug (IND) approval to safely proceed with the use of Timoptic-XE® (timolol maleate ophthalmic gel forming solution, 0.5% for use on DFUs, IND number 122399).

#### Drug management and record keeping

The Sacramento VA research pharmacist will receive and manage both the non-biologically active gel (placebo) and the Timoptic-XE®. The pharmacist will randomize the patients between the two groups, using the website Randomization.com [57], and will dispense either the Timoptic-XE® or non-biologically active gel hydrogel to the patients according to the arms to which they have been randomized. The research pharmacist will obtain both the study drug and placebo medication from their respective manufacturers, and the study team will provide the research pharmacy with clear empty identical dispensing bottles covered with amber bags to protect the drug from light. The pharmacist will employ Good Clinical Practice methods when transferring the active or placebo study drugs into the dispensing bottles. Once transferred, the bottles are placed into amber zip lock bags and labeled with the appropriate drug code and auxiliary labels. Subjects will be provided with 12 weeks’ supply according to their individual randomized unique identifier to ensure accurate storage and dispensing records. The unblinded research pharmacist will keep a record of the drug and patient treatment group assignment.

The recommended Timoptic-XE® ocular dosage is 0.25 mg/day (1 drop in each eye once a day) [49, 58, 59]. The average exposed ocular surface is about 3 cm^2^ [32]. Based on the studies using timolol topically for ulcerative hemangiomas [46–54, 56, 60] and the numerous case studies of topical timolol on chronic wounds [39, 40, 61, 62], the maximum dosage used in this study will be 3 drops/3 cm^2^/day, which is equivalent to 0.75 mg/3cm^2^/day (Table 3). For study patients, depending on the size of the DFU, see Table 3, either topical timolol or placebo drug (non-biologically active gel) will be administered as one drop daily for < 0.5cm^2^ to > 0.5–1.9 cm^2^, two drops daily for wound size > 2–2.9 cm^2^, and three drops daily for anything > 3 cm^2^ (maximum dose). Depending on the size of the wound, the research pharmacist will dispense one or two bottles (with either Timoptic-XE® or placebo) for the 12 weeks’ supply as described in the table below.

**Table 3:** Timolol dosage management

Wound size (cm^**2**^)	Number of drops	Timolol dosage (mg/day)	Timolol dosage (mL/day) Note: 1 drop = 0.05 ml	Dosage for 12-week supply (ml)	Number of bottles (timolol or placebo) dispensed to patient for 12-week supply (1 bottle = 5 ml)
**< 0.5**	1	0.25	0.05	4.2	1
**> 0.5–0.9**	1	0.25	0.05	4.2	1
**> 1.0–1.9**	1	0.25	0.05	4.2	1
**> 2.0–2.9**	2	0.5	0.1	8.4	2
**> 3.0**	3	0.75	0.15	12.6	3

#### Selection of treatment site

For subjects presenting with multiple wounds, the largest wound will be the index ulcer, and will be greater than 3 cm distance from any other ulcer. The index ulcer will be assessed and treated prior to all non-index ulcers to avoid cross-contamination. All non-index ulcers will be treated per physician discretion. The subject will be followed weekly in clinic visits and the evaluation and assessment of the ulcers will be performed as described below.

#### Informed consent and enrollment

This study will be conducted exclusively on veterans within the VANCHCS with a documented diagnosis of diabetes who have had a foot ulcer for at least 4 weeks. Patient recruitment and obtaining consent will be performed by the research team, which includes research investigators, a research coordinator, and a wound research fellow. Any information collected in this study will remain confidential and no identifying information will be released during this study. HIPAA guidelines will be followed. A unique study identifier (ID) will be assigned to participants and no personal information will be linked to patients.

Patients who meet the eligibility criteria, as described in Table 1, will be approached to participate in the study. Any potential subject that agrees to join the study will be provided with VA IRB approved informed consent/HIPAA documents. Research staff will provide a detailed description of the study purpose, procedures, duration, and potential risks, as described in Table 4. Each subject will be provided with adequate time to consider the information and ask any questions before signing the informed consent document. Subjects will be provided with a copy of their signed/dated informed consent and another copy will be kept as part of the subject’s research study record; those subjects will be considered enrolled in the clinical trial, meeting all inclusion and no exclusion criteria as they enter the screening phase.

**Table 4:** Potential risks and adequacy of protection from risks

Physical risk: low to moderate	
● Blood draw: possible pain associated with needle stick, ecchymosis, or phlebitis	
● Randomization: subject assignment to the therapeutic group may be less beneficial and associated with more adverse events than standard of care	
● Other possible risks: osteomyelitis, cellulitis, dermatitis, eczema, rash, allergic reaction	
● Potential severe but rare risks: cessation of heartbeat and respiratory failure	
Psychological risk: low	
● However, no direct causal relationship has been established to therapy with Timoptic-XR®. These rare AEs may include depression, confusion, anxiety, disorientation, nightmares, somnolence, insomnia, diminished concentration, hallucinations	
Social risk: low	
● Subjects are required to visit the clinic weekly, which may take time away from other activities and tiredness while waiting for weekly office visits	
Economic risk: moderately low ● Cost of travel to and from weekly appointments. Due to frequency of visit, subjects may lose time from work. Note: subjects would be required to travel for weekly visits regardless of whether the subject had been enrolled in the study, since standard of care typically requires weekly visits	
Physical risk: low	
● All subjects in the study will have their personal information confidentially secured in locked filling cabinets and with protected passwords in a computer database. Access is strictly limited to authorized individual (principal investigator, research staff, or other regulatory authorities such as representatives of FDA or IRB).	

#### Randomization

The Randomization.com website will be used for simple randomization allocation. It is a website programmed to perform randomization schemes through randomization plan generators. The specific randomization scheme for the BAART study randomizes 48 subjects to an active component and a placebo component. If the study exceeds the expected 48 targeted subjects for enrollment, the program can be altered so that the same scheme is generated to randomize additional subjects. Each subject will be given a unique identifier with no personal information linked after assignment according to a randomly computer generated listed of two repeated numbers for placement into either group A or group B. The research pharmacist will dispense either Timoptic-XE® or the non-biologically active gel hydrogel to patients according to their randomized arm. Subject demographics will not be recorded until this assignment is complete. The patients will be blinded to study drug treatment.

### Study schematic

#### Enrollment

All participants are screened for eligibility based on the inclusion and exclusion criteria. The eligible participants will be called by the research coordinator and invited to participate in the study (Fig. 2).

#### Screening phase–2 weeks’ duration (visits 1–2/weeks 1–2)

Subjects will be screened to determine if they meet the inclusion and exclusion criteria requirements, and those with any clinical conditions that may contraindicate the use of the study drug are excluded from the study. Table 5 describes the workup performed in order to determine eligibility for this study. Subjects will receive comprehensive training on protocol-specified treatment application in order to determine their ability to comply with this study. Each subject will receive SOC as described previously (Table 2). At the completion of the 2-week screening phase, subjects who experience more than 40% change in ulcer size will be exited from the study prior to randomization (since they are not difficult-to-heal ulcers).

**Table 5:** Screening assessments and pre-treatments

Demographic information	Gender, age, race
Medical history	Medical problems, surgeries, trauma, history of previous ulcers, amputations, characteristics, and duration
Comprehensive history and physical exam	Vital signs, height, weight, body mass index
General health and lifestyle	Smoking history, alcohol, drug abuse
Lower extremity exam	Vascular—pedal pulses, color of skin, temperature, edema. Dermatological—clinical description of the ulcer, fungal infection of skin and/or nails, skin integrity (calluses, dryness). Musculoskeletal—foot deformities such as bunion, hammertoe, bony prominence, fat pad atrophy, altered gait. Neurological—absence or presence of sensation with 5.07/10 Semmes-Weinstein monofilament, reflexes
Non-invasive vascular study	Ankle–brachial systolic pressure (ABI) and toe-brachial systolic pressure (TBI). In order to meet criteria, ankle–arm index must be equal to or greater than 0.8 and less than 1.4 or a toe-arm index is equal to or greater than 0.6
Foot ulcer history	Location, length of time, treatments used, pain, etiology of ulcer
Laboratory	Hematology, chemistry, EKG, microbiology and pathology HbA1c, pregnancy test (for women of childbearing ages), LFT, ESR, CRP, and albumin
Radiological imaging	Plain foot and/or ankle films for baseline
Health—quality of life surveys	VR-36 Health Survey and the Lower Extremity Functional Scale Outcome Questionnaire
Debridement and specimen collection	Sharp debridement of ulcer will be performed per standard method. A small sample will be collected for microbiology (gram stain, cultures/sensitivities, and fungal) and pathology
Photographs	Before and after debridement using Silhouette Mobile™ ulcer tracing, surface area calculation
Dressings	Non-adhesive dressing (Adaptic® or Mepitel®) over wound bed, covered by dry dressings
Off-loading	Shoes will be given, modified offloading insert (trilaminar plastazote) as determined appropriate at the discretion of the clinician

#### Active phase–12 weeks (visits 3–15/weeks 3–15)

##### Randomization visit (visit 3/week 3)

Subjects who successfully meet the study requirements and complete the screening phase will be randomized into either group A (Timoptic-XE® plus SOC) or group B (non-biological gel plus SOC). Subjects in group A will undergo the same SOC, including wound care and offloading modality, as individuals in group B. The placebo medication will be applied to the wound daily with the same dosage as above.

##### Treatment phase (visits 4–14/weeks 4–14)

Subjects will be evaluated and receive treatments on a weekly basis (7 days ± 2 days). The investigator (physician) will determine the time of wound closure, as defined by “skin re-epithelialization” without drainage or dressing requirements confirmed at two consecutive study visits, 2 weeks apart. If the ulcer remains healed at the second confirmatory visit, the subject will proceed to the follow-up phase, i.e., these patients will skip any remaining visits in the active phase.

Blood samples will be taken from each subject at weeks 4, 8, and 12 to determine plasma timolol level. We do not anticipate blood levels in our patients to be higher than those seen in patients who receive Timoptic XE® gel for ocular indication (normal range 0.3–0.5 ng/ml) [63]. Subjects in whom the timolol level is 0.7 ng/ml or higher will be removed from the study.

##### Study primary endpoint (visit 14/week 14) and 2nd confirmatory visit (visit 15/week 15)

At the end of study treatment, the ulcer will be assessed for healing characteristics to determine if it has completely epithelialized; if there are concerns, further workup will be obtained and addressed accordingly. The subjects will also complete the health-related quality of life survey or the VR-36 Health Survey and the Lower Extremity Functional Scale Outcome Questionnaire.

#### Follow-up phase (visit 16–19/week 19–31)

Subjects will be seen for follow-up visits at monthly intervals for 4 months. Those subjects who have unhealed wounds at completion of the final study visit will continue with SOC at a regular wound clinic. At each follow-up visit an evaluation of the durability of wound closure of the study ulcer will be assessed by the clinician. Digital imaging of ulcer location of non-healed ulcer, study staff will be obtained pre- and post-debridement images. Any changes in concomitant medications, adverse events, or compliance with offloading will be recorded.

#### Withdrawal

Subjects may request to withdraw from the study at any time. The investigator may also remove a subject if it is determined that the subject develops serious adverse event (SAE) related to the study. A SAE is defined by the FDA as any adverse drug event that results in any of the following outcomes: death, life-threatening adverse events, inpatient hospitalization, or prolonged existing hospitalization for more than 24 h [64]. Intention-to-treat analysis will be performed. Subjects who are withdrawn from the study will be followed via patient chart reviews from their regular wound care visits. Those subjects that have undergone treatment will be followed and documented until the end of the study. If there is more than 15% loss to follow-up, investigators will attempt to recruit additional subjects.

#### Non-compliance

During the screening period, one-to-one training will be given to ensure that the subjects are able to apply the medication and proper dressing as per the protocol. A unique challenge may be related to the application of the offloading device. We propose to prevent this potential problem by providing the opportunity for subjects to return to the clinic the same day if they feel that the offloading device needs to be adjusted prior to the next scheduled appointment. We will provide subjects with monetary compensation at the end of the active phase, and then the remaining compensation will be given at the end of the study (upon completion of the follow-up phase).

#### Loss to follow-up

Our clinical research staff will phone each patient prior to their appointment days to keep communication lines open. If patients are unable to visit our clinic for a particular scheduled visit, our staff will reschedule to accommodate his/her schedule in a ± 7-day window and perform all study-related functions for that visit.

#### Blinding/breaking the blind

The active and placebo study drugs will be received from the manufacturer by the research pharmacist, who will repackage them in coded dropper dispenser bottles. Since the drug and placebo will be repackaged into identical dispensers, investigators, research staff members, including the research coordinator, research fellow, sponsors, and study subjects, will be blinded to the study treatment.

In the event of any SAEs related to the study, the investigator will break the blind [65]. In addition, the DSMB will regularly review the obtained research data and will trigger modifications to the trial or the management of an individual subject should SAEs arise. The DSMB, located at Hines VA Hospital, 60,141, IL, USA, is independent of the sponsor, competing interests, and of our local facility.

#### Adverse events

All adverse events (AEs) that occur during the course of the study will be noted and documented by type, seriousness, intensity/grade, relationship to diabetes, and the unique therapy. All AEs will be reported to the principle investigator. Each week, the study investigator will review the AE forms from the previous week for any new or continuing events. A study participant may have their medication discontinued or may be withdrawn from the study if the medically responsible investigator determines it is the best decision in order to protect the safety of the participant.

At each visit, the investigator will clinically assess the subject’s wound for infection. The presence of moderate or severe signs/symptoms of inflammation [66] will prompt further laboratory evaluation (i.e., hematology, wound culture). Infected ulcers will result in study exit and an appropriate documentation of the AEs and treatment per protocol. Also, the DSMB will monitor the study and provide guidance if AEs are noted. The DSMB will regularly review the obtained research data and will trigger modifications in the trial or in the management of an individual subject should SAE arise.

### Analysis

#### Sample size estimation and randomization

From previous studies [11, 18, 19], we expect that about 20% of the study subjects will heal in the control arm of our study by the 12th week of care. We estimate that the experimental treatment will improve this outcome with 63% of the study subjects healing by the 12th week of care (a healing rate close to that reported in the most recent case series of topical use of timolol on chronic wounds [67]). We will perform simple randomization of the subjects using electronic randomization for treatment assignment since our target enrollment population is fairly homogenous.

The sample size calculation requires the enrollment of 48 subjects (24 subjects in each arm) to provide statistical power of 80% to detect a difference of 43% (63% − 20%) in the rate of ulcer healing between the two arms using two-sided Fisher’s exact test at a significance level of 5%. It is estimated that the overall attrition will be 35%. Specifically, 10% of the subjects who are enrolled will be exited from the study prior to randomization, 10% will not meet the primary endpoint analysis inclusion requirement, and 15% will not complete the study due to other factors such as treatment failure, loss to follow-up, adverse events, clinical/safety issues, and/or non-compliance. We will track the various reasons for study exit. Therefore, 138 subjects (69 subjects in each arm) will be recruited into the study. After taking into account an overall attrition of 35%, a total of 48 patients will be enrolled in the study. The consulting statistician will review implementation and compare major baseline demographic and prognostic characteristics to ascertain that randomization was successful. The power analysis was formulated using the STPLAN version 4.5 (2010) [68] with the input of the study consultant biostatistician. When study is at the midpoint, interim analysis will be performed to assess for the primary outcome for the two study treatment groups.

#### Data management plan

The primary outcome analyses will be performed on an intent-to-treat basis. The analysis of subjects will be done per study group regardless of missing data from subjects who did not complete the study. We will impute missing data by using multiple imputation techniques in the analyses.

All of the patient’s data will be securely stored in locked cabinets per the VA protocol and in secure VA servers that provide centralized file storage and backup, and they will only be accessible by the research staff. The final data and results from the study will be shared via publication. Electronic datasets will be de-identified and anonymized. These datasets will be maintained locally on VA protected servers until enterprise-level resources become available for long-term storage and retrieval.

## Discussion

This is a clinical translation study, moving the investigators’ pre-clinical laboratory research into the first randomized clinical study. Our previous work has demonstrated that stress-catecholamines such as epinephrine and norepinephrine, βAR agonists, impair wound healing as evidenced by decreased keratinocyte migration and a decrease in keratinocytes’ ability to heal wounds in vitro [23, 27, 28, 31, 33, 34]. Interestingly, not only can stress-induced elevation of systemic catecholamines impair healing [69, 70], but as we and others have shown, the keratinocytes themselves can generate epinephrine [25, 71] and significant levels of catecholamines are present within the immediate vicinity of the wound [24]. Conversely, our laboratory investigations have shown that βAAs increase keratinocyte migration speed (a surrogate for epithelial healing), the healing of scratch wounds in confluent keratinocyte cultures [31], and wound epithelialization in vivo in a number of animal wound models [24, 26, 28, 72]. Additionally, in a human skin ex vivo burn wound model, cultivation with βAA (timolol) improves wound re-epithelialization relative to the culture medium control. Further, our studies have shown that catecholamines increase neutrophil dwell time in the wound, delaying healing in an animal wound model, and treatment with βAA reverses this, restoring healing time to normal [73]. We and others have also documented and reported on cases of venous leg ulcers and other chronic wounds in humans that have improved healing after topical timolol application [38, 61, 62, 67]. These multiple laboratory studies and anectdotal clinical reports indicate that βAAs (timolol) promote wound healing and shorten time to healing; thus, a clinical trial is the next logical step to determine if this approach is truly efficacious in the clinical setting of DFUs.

We hypothesized that subjects with DFU treated with a βAA (timolol) will have more rapid complete wound closure compared to those in the SOC group. The null hypothesis is that there is no difference in the proportion of subjects with complete wound closure of DFUs between the two treatment arms.

The Cochrane Wounds Group has indicated that the healing rates and healing time commonly reported in the majority of wound studies and clinical trials area deemed appropriate primary outcomes [74]. In fact, most studies have demonstrated that the proportion of ulcers healed at 12 weeks of treatment is an appropriate outcome measure [18, 19]. Thus, for our study purposes, the primary endpoint is complete ulcer closure as defined by “skin re-epithelialization without drainage or dressing requirements” by week 12 (end of the active phase of the study).

We will also assess secondary endpoints that will focus on measurements obtained throughout the clinical trial, including change in percentage reduction in wound size between the two study arms, calculated as difference in cm^2^ of the randomization measurement after 12 weeks of the active phase and after the follow-up phase. We will consider patient’s quality of life using the VR-36 Health Survey, the Lower Extremity Functional Scale [39], and the Charlson Comorbidity Index [40].

AEs will be assessed for safety parameters that specifically include SAEs, hospitalization, study exit, unanticipated adverse device effects, and therapy-related incidences. We will analyze and compare the data between these two treatment arms, including AEs at an incidence of 1% or greater, adverse reactions to the test therapy at an incidence of 1% or greater, incidence of immediate reactions, incidence of local and systemic reactions, incidence of osteomyelitis, incidence of wound infection, and incidence of SAEs or unanticipated adverse device effects. Potential confounding variables include Basic Metabolic Index (BMI), wound size (which we will adjust for using the subcategories < 0.4 cm^2^, > 0.5–0.9 cm^2^, > 1.0–1.9 cm^2^, > 2.0–2.9 cm^2^, > 3.0 cm^2^), as well chronicity of the wound by year (adjusted for using < 1 year, > 1–3 years, > 3–5 years, > 5 years, > 10 years). Safety data collected from the clinical trial will significantly contribute toward establishing the safety profile of topical βAA use in DFUs.

There may be several potential limitations to this study involving study subjects’ demographics. As this study occurs solely at veteran medical centers, there may be bias for recruiting mainly veteran male patients. Compared to the US general population (49% male, 50% female), females represent only 9% of the veteran population nationwide [9, 75]. Similarly, there may also be bias toward recruiting middle-age, elderly, and white veterans at the Veteran Health System [76], but it should be noted that DFUs are more common between the ages of 60 and 80 years [2, 4, 5, 8, 14–16, 55, 77].

## Trial status

Protocol date and version: December 5, 2019, version 4.

Patient recruitment began in August 2018 at the Sacramento VA Medical Center, Mather, CA, USA. Study enrollment and recruitment are estimated to be completed within 4 years.

## Data Availability

A copy of the informed consent or dataset analyzed during the current study are available from the corresponding author on reasonable request.

## Abbreviations

DFU: Diabetic foot ulcer
βAA: Beta-adrenergic antagonists
βAR: Beta-adrenergic receptors
MSC: Mesenchymal stem cells
IRB: Institutional review board
SOC: Standard of care
FDA: Food and Drug Administration
ABI: Ankle brachial index
DSMB: Data and safety monitoring board
AE: Adverse events
ANOVA: Analysis of variance
QOL: Quality of life
VR-36: Veterans Rand-36
BMI: Basic Metabolic Index
VA: Veterans Affairs
